# The association between articular calcium crystal deposition and knee osteoarthritis, joint pain and inflammation: a cross-sectional study

**DOI:** 10.1007/s00256-025-04904-7

**Published:** 2025-03-14

**Authors:** Camilla Toft Nielsen, Marius Henriksen, Cecilie Laubjerg Daugaard, Janus Uhd Nybing, Philip Hansen, Felix Müller, Henning Bliddal, Mikael Boesen, Henrik Gudbergsen

**Affiliations:** 1 Department of Radiology, Copenhagen University Hospital - Bispebjerg and Frederiksberg, Bispebjerg Hospital, Entrance 7A, Nielsine Nielsens Vej 41A, 2400 Copenhagen, NV Denmark; 2 The Parker Institute, Copenhagen University Hospital - Bispebjerg and Frederiksberg, Frederiksberg Hospital, Nordre Fasanvej 57, Road 8, Entrance 19, 2000 Frederiksberg, Denmark; 3 Department of Radiology, Copenhagen University Hospital - Herlev and Gentofte, Herlev Hospital, Borgmester Ib Juuls Vej 17, Entrance 4, 4Th Floor, E2, 2730 Herlev, Denmark; 4 Department of Public Health, Centre for General Practice, University of Copenhagen, Øster Farimagsgade 5, Build. 24 Q, 1St Floor, 1353 Copenhagen K, Denmark

**Keywords:** Osteoarthritis, Crystal arthropathy, CPPD, BCP, Inflammation, Pain, Imaging, MRI, CT

## Abstract

**Objective:**

To explore in a cross-sectional fashion if overweight individuals with knee osteoarthritis (OA) and intraarticular calcium crystal (CaC) deposits experience more knee joint inflammation and knee pain compared with individuals without CaC deposits.

**Subjects and methods:**

We used pre-randomization imaging data from an RCT, the LOSE-IT trial. Participants with knee OA (clinical diagnosis of knee OA and KLG 1–3) had CT and 3 T MRI of the index knee. CaCs were assessed on CT using the Boston University Calcium Knee Score (BUCKS). The pain subscale of the Knee Injury and Osteoarthritis Outcome Score (KOOS) was used to assess knee pain and to estimate joint inflammation we used static and dynamic contrast-enhanced (DCE) MRI. An independent sample *t*-test was used to test for a significant difference in KOOS-pain and Analysis of Covariance (ANCOVA) models to test for differences in the static and DCE-MRI variables between the two groups.

**Results:**

Of the 158 participants with KOOS-pain available, 19 (12%) had CaC deposits, and of the 115 participants with MRI available, 13 (11.3%) had CaC deposits. We did not find a significant difference in mean KOOS-pain between the two groups; the mean difference was − 2.2 points (95%CI, − 10.86, 6.45). None of the MRI variables were associated with the presence of CaC deposits. Between-group differences were small for all MRI variables, with standardized mean differences ranging from small to medium (0.31–0.56).

**Conclusion:**

In individuals with knee OA, we did not find an association between intraarticular CaC deposits and an increase in knee joint inflammation or knee pain.

**Supplementary Information:**

The online version contains supplementary material available at 10.1007/s00256-025-04904-7.

## Introduction

Calcium crystal (CaC) deposition is found in the cartilage, menisci, and joint capsule of the knee in some patients with osteoarthritis (OA). CaCs are predominately comprised of calcium pyrophosphate dihydrate (CPP) and basic calcium phosphate (BCP) crystals [1]. Their role in OA is poorly understood. The reported prevalence of CaC deposits in patients with OA varies depending on the method of crystal detection, and it increases with OA severity [2]. Synovial fluid (SF) analysis is the gold standard for detecting CPP crystals, whereas more specialized methods, such as Raman spectroscopy, are needed to detect BCP due to their small size. In a cohort of individuals with end-stage knee OA, CPP crystals were found in 30% of the SF analyzed, but only 31% of the SF-positive samples showed chondrocalcinosis on radiographs [3]. Compared to radiographs, Computed Tomography (CT) has a higher sensitivity for crystal detection [4].

CaC depositions in the cartilage and meniscus have been associated with symptomatic and structural disease progression in patients with knee OA [5, 6]. In vitro, CaCs activate pro-inflammatory pathways, resulting in increased interleukin-1β, nitric oxide, prostaglandin E2, and matrix metalloproteinase-13 [7, 8]. Reports on the inflammatory effects of CaCs in in vivo human studies are more conflicting; some studies suggest an association between CaC deposits and increased knee joint inflammation while others do not [2, 9, 10].

Magnetic resonance imaging (MRI) is a non-invasive method for evaluating intraarticular inflammation. Non-contrast-enhanced MRI cannot distinguish synovitis from effusion and contrast-enhanced (CE) MRI is better at evaluating synovial inflammation [11]. Dynamic contrast-enhanced (DCE) MRI is rapid T1-weighted sequences obtained before, during, and after intravenous injection with a gadolinium-based contrast medium. It reflects the perfusion and permeability of the tissue. DCE-MRI is valuable in evaluating synovial inflammation in OA research.

OA presents as a diverse and complex condition, manifesting in various forms and subtypes [12]. Understanding the role of different pathological tissues or pathways complicit in OA development and progression is important. CaCs might be a treatment target in OA disease-modifying drug trials in this subgroup of patients. However, more research is needed to clarify the effect of CaCs on both patient symptoms and structural changes in OA. With this exploratory analysis, we test the hypothesis that individuals with overweight and knee OA with CaC deposits have more knee joint pain and knee joint inflammation compared to individuals without CaC deposits.

## Subjects and methods

The “Liraglutide 3 mg for Knee Osteoarthritis (LOSEIT)” trial (ClinicalTrials.gov identifier: NCT02905864) was a randomized controlled trial (RCT) designed to test the efficacy and safety of liraglutide, a glucagon-like peptide-1 receptor agonist, in patients with knee OA and overweight. Participants between 18 and 75 years, with a clinical diagnosis of knee OA, according to the American College of Rheumatology (ACR) criteria; Kellgren–Lawrence Grade (KLG) 1–3; and body mass index (BMI) ≥ 27 kg/m^2^, were included in the LOSEIT trial. Participants were enrolled in one OA outpatient clinic from November 2016 to September 2017. A detailed description of the LOSEIT trial has been published elsewhere [13]. All study participants signed informed consent prior to participation in the LOSEIT trial. Participants underwent an 8-week weight loss intervention from enrolment to randomization (week − 8 to 0) followed by a 52-week drug intervention period (week 0 to 52). In this cross-sectional study, we only use data from the pre-randomization period. The Knee Injury and Osteoarthritis Outcome Score (KOOS) was recorded, and a 3 T MRI (Verio®, Siemens, Erlangen, Germany) of the target knee was performed. In addition, participants underwent CT (Somatom Definition Edge®, Siemens, Erlangen, Germany) of both knees. Knee joint pain was assessed by the KOOS-pain subscale at week − 8 and knee joint inflammation was assessed on MRI of the target knee also at week − 8. CaCs were assessed on CT of the target knee at week 0, i.e., after weight loss intervention.

Informed consent was obtained from all participants included in the study. The LOSEIT trial was approved by the Local Health Research Ethical Committee and conducted in accordance with the Helsinki Declaration.

### Boston university knee calcium score

A modified version of the Boston University Knee Calcium Score (BUCKS) [14] was used to quantify intraarticular mineralization on CT (in-plane resolution, 0.6 × 0.6 mm; slice thickness, 1 mm; tube voltage, 140 kV). One resident with 3 years of experience in rheumatology and radiology (CTN) performed BUCKS scoring, supervised by a senior musculoskeletal (MSK) radiology consultant and professor (MB). An ordinal score of 0–3 was used to score the presence of mineralization in the hyaline cartilage, the lateral meniscus, and the medial meniscus. A dichotomous score of 0 for the absence and 1 for the presence of mineralization in the joint capsule was used. Mineralizations in the ligaments and vessels were excluded as we believe they represent different disease entities; calcific tendonitis and atherosclerosis, respectively. Participants were classified as OA with CaC if they had a BUCKS ≥ 1 in any sub-region. Case examples and a description of the ordinal score used in the BUCKS can be found in Fig. S1 in the supplementary material.

### Knee injury and osteoarthritis outcome score

Knee joint pain was assessed by the pain subscale of the Knee Injury and Osteoarthritis Outcome Score (KOOS) [15, 16]. KOOS is an extension of the Western Ontario and McMaster University Osteoarthritis Index (WOMAC) and a validated self-reported questionnaire. It is used to evaluate symptoms and function in individuals with knee injury and/or knee OA, both short- and long-term symptoms. The pain subscale consists of nine items answered by a 5-point Likert scale from 0 (no pain) to 4 (extreme pain). The score is transformed into a percentage score with 100 indicating no pain and 0 indicating extreme pain.

### MRI analyses

The MRI analyses were performed by a resident in radiology with 3 years of experience in MSK radiology (CLD) supervised by a senior MSK radiology consultant and professor (MB). A detailed description of the MRI analyses has been published elsewhere, including intra-reader reliability which ranged between 0.87 and 1.00 for all MRI variables [17]. Below is a short description of the MRI variables used in this study.

#### Static MRI

MRI in OA Knee Score (MOAKS) was analyzed on a non-contrast-enhanced 2D PD-weighted FS sequence. In order to have one score for each static MRI variable, the Hoffa-synovitis score and the effusion-synovitis score were summed to one MOAKS-synovitis score [18]. Although conventionally scored on non-contrast-enhanced sequences, The Boston–Leeds Osteoarthritis Knee Score (BLOKS) effusion score [19] was analyzed on contrast-enhanced (CE) axial post-gadolinium contrast 3D GRE T1-weighted VIBE sequences in order to have a score distinguishing the effusion from the synovium and not analyzing them collectively as in the MOAKS score. The same CE axial post-gadolinium contrast 3D GRE T1-weighted VIBE sequence was used to score the 11-point whole-knee synovitis score (CE-synovitis) as proposed by Guermazi et al. [20].

#### Dynamic contrast-enhanced MRI

**Fig. 1:**
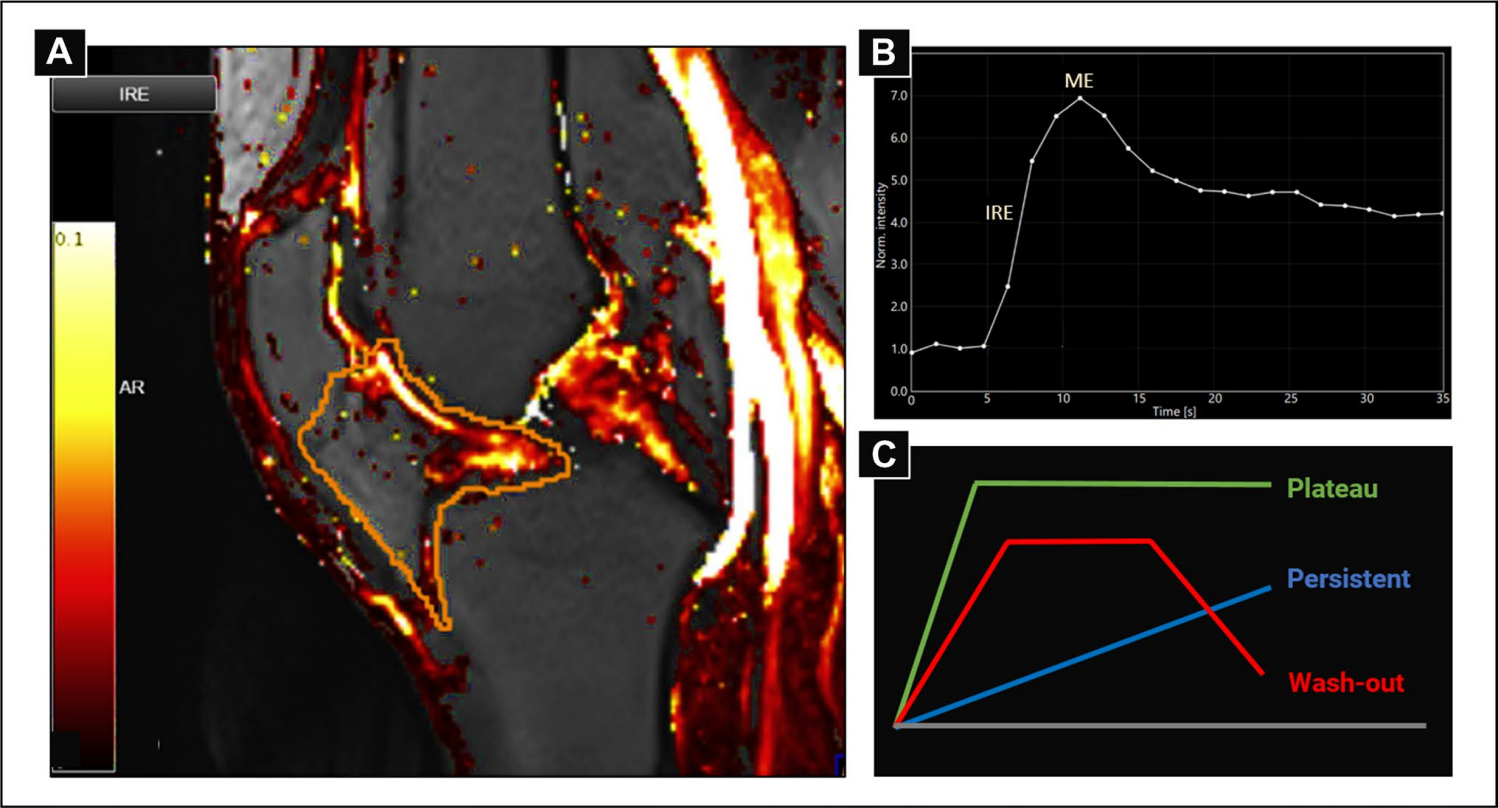
**a** DCE-MRI parametric map of the initial rate of enhancement; brighter colors indicate higher values. **b** Time-intensity curve (from the popliteal artery). **c** Schematic illustration of the different perfusion patterns. IRE Initial rate of enhancement, ME maximum enhancement. **a** and **b** are adapted from the work of C. Daugaard^17^, with permission from the author

DCE-MRI is a serial acquisition of T1-weighted sequences during intravenous injection of a contrast medium. The change in signal intensity (contrast uptake) in the tissue can be plotted against time, resulting in a Time Intensity Curve (TIC), Fig. 1b. Furthermore, the uptake pattern for each individual voxel is assigned to one of four perfusion patterns: no enhancement, persistent, plateau or wash-out, Fig. 1c. The software Dynamika® v. 5.2.2 (Image Analysis Group Ltd., London, UK, www.imageanalysis.org.uk) was used for the analyses. Regions of interest (ROIs) were drawn around the infrapatellar fad pad on each image slice, Fig. 1a, and then collapsed to one volume of interest (VOI), with only enhancing voxels contributing to the perfusion variables. Each DCE-MRI variable was calculated in a voxel-by-voxel analysis, i.e., as a mean of all the enhancing voxels within the VOI. The analyses were done using the semi-quantitative heuristic approach, where the DCE-MRI variables are derived directly from the TIC. We used three DCE-MRI variables: Initial Rate of Enhancement (IRE), the initial upslope on the TIC, Maximum Enhancement (ME), the highest mean signal intensity value, Fig. 1b; and Most Perfused Voxels (Nvoxel), the volume of voxels with the highest perfusion pattern (plateau and wash-out), Fig. 1c. IRE and ME are surrogate measures of the degree of tissue perfusion, while Nvoxel represents a volume of the most perfused voxels (i.e., the volume of synovitis). In addition, two composite scores were calculated; IRE x Nvoxel and ME x Nvoxel, reflecting both the volume and the degree of perfusion. IRE and ME have been shown to be highly correlated with histological inflammation [21, 22], while ME, IRExNvoxel, and MExNvoxel have been associated with self-reported joint pain [22–24].

### Statistical analysis

The statistical analyses were all predefined in the statistical analysis plan. All statistical analyses were conducted using the statistical program R, v. 2022.07.01 + 544 (https://www.r-project.org/). The analysis population included all participants with complete CT and outcome measures, existing case population, i.e., no imputation for missing data. To test if there was a difference in KOOS-pain between participants with and without CaC deposits, we used an independent sample *t*-test. To test if there was a difference in the DCE-MRI and static MRI variables between participants with and without CaC deposits, we used an Analysis of Covariance (ANCOVA) model adjusted for age and KLG as covariates. Standardized mean differences (Cohen’s *d*) are reported with interpretation intervals as suggested by Cohen (small *d* = 0.2, medium *d* = 0.5, large *d* = 0.8) [25]. Statistical significance was accepted at *p* < 0.05. Diagnostic plots were used to visually assess the assumptions for the ANCOVA model.

## Results

**Fig. 2:**
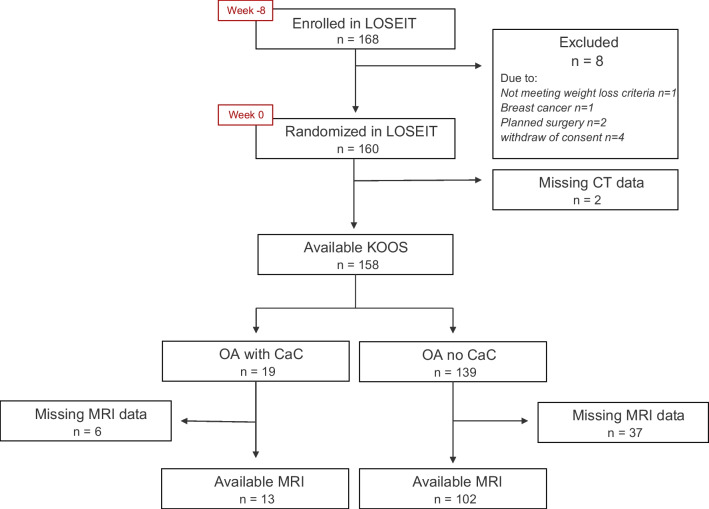
Flow diagram of study inclusion

Of the 168 participants included in the LOSEIT trial, CT scans were available for 158 participants. All 158 participants had KOOS-pain available, and 115 participants completed an MRI. Cases where MRI was not available were due to knee circumference above 53 cm, the size limit of the MRI-coil, an eGFR below 60 ml/min/1.73 m^2^, allergy to the MRI contrast agent, or in four cases, significant artifacts. Of the 158 participants with KOOS-pain available, 19 (12%) had CaC deposits on CT, and of the 115 participants with MRI available, 13 (11.3%) had CaC deposits on CT, Fig. 2.

Participant characteristics are presented in Table 1. Participants were comparable on baseline characteristics including age, sex distribution, and BMI. However, a higher percentage of participants in the OA with CaC group had KLG 3 compared to the percentage of participants in the OA no CaC group. The mean (SD) age was 59.2 (± 10.2) years and 64.6% were female. Mineralizations were present in the cartilage of ten participants and the meniscus of ten participants. Only two participants had mineralizations in the joint capsule.

**Table 1.** Demographic characteristics and change in KOOS and MRI variables between groups

Characteristics	OA no CaC (*n* = 139)	OA with CaC (*n* = 19)	Total (*n* = 158)	Difference between groups (95% CI)	^*†*^*P* − value	SMD (95% CI)
Age	59 (± 10.3)	60.4 (± 9.6)	59.2 (± 10.2)			
Female, *n* (%)	90 (64.7)	12 (63.2)	102 (64.6)			
BMI	36.4 (± 5.4)	35.3 (4.8)	36.3 (± 5.4)			
KLG 1, *n* (%)	20 (14.4)	3 (15.8)	23 (14.6)			
KLG2, *n* (%)	65 (46.8)	6 (31.6)	71 (44.9)			
KLG3, *n* (%)	54 (38.8)	10 (52.6)	64 (40.5)			
*BUCKS*						
Cartilage, *n*		10				
Meniscus, *n*		10				
Joint capsule, *n*		2				
*KOOS-pain*						
KOOS-pain	63.7 (± 16.4)	65.9 (± 17.2)	64.0 (± 16.4)	− 2.2 (− 10.86; 6.45)	0.603	− 0.13 (− 0.61; 0.35)
**Characteristics** *For patients with MRI available*	**OA no CaC** (*n* = 102)	**OA with CaC** (*n* = 13)	**Total** (*n* = 115)	**Difference between groups** (95% CI)	^***‡***^***P* − value**	**SMD**
Age	59.9 (± 10)	57.5 (± 9.5)	59.6 (± 10)			
Female, *n* (%)	64 (62.7)	7 (53.8)	71 (61.7)			
BMI	34.8 (± 3.9)	33.6 (± 3.2)	34.6 (± 3.8)			
KLG 1, *n* (%)	17 (16.7)	1 (7.7)	18 (15.7)			
KLG 2, *n* (%)	51 (50.0)	5 (38.5)	56 (48.7)			
KLG 3, *n* (%)	34 (33.3)	7 (53.8)	41 (35.7)			
*Dynamic MRI variables*						
IRE (%/s)	0.0375 (± 0.020)	0.0441 (± 0.028)	0.038 (± 0.021)	− 0.005 (− 0.017; 0.007)	0.438	− 0.31 (− 0.89; 0.27)
ME	1.63 (± 0.239)	1.77 (± 0.300)	1.650 (± 0.249)	− 0.114 (− 0.252; 0.023)	0.103	− 0.56 (− 1.14; 0.03)
Nvoxel (ml)	13.8 (± 6.85)	17.0 (± 6.08)	14.2 (± 6.82)	− 2.430 (− 6.12; 1.27)	0.195	− 0.47 (− 1.05; 0.11)
IRE x Nvoxel	0.583 (± 0.531)	0.804 (± 0.652)	0.608 (± 0.547)	− 0.174 (− 0.482; 0.134)	0.265	− 0.40 (− 0.98; 0.18)
ME x Nvoxel	23.4 (± 13.5)	31.0 (± 14.0)	24.3 (± 13.7)	− 6.160 (− 13.6; 1.24)	0.102	− 0.56 (− 1.14; 0.02)
*Static MRI variables*						
MOAKS synovitis	2.47 (± 1.43)	3.00 (± 1.22)	2.53 (± 1.42)	− 0.398 (− 1.2; 0.406)	0.329	− 0.37 (− 0.95; 0.21)
BLOKS effusion	0.794 (± 0.937)	1.31 (± 0.855)	0.852 (± 0.939)	− 0.411 (− 0.955; 0.133)	0.137	− 0.55 (− 1.13; 0.03)
CE-synovitis	4.40 (± 3.25)	5.54 (± 3.41)	4.53 (± 3.27)	− 0.750 (− 2.6;1.1)	0.424	− 0.35 (− 0.93; 0.23)

Values are mean (SD) unless otherwise stated in the table

*SMD* standardized mean difference (Cohen’s *d*), *CaC* calcium crystals, *BUCKS* Boston University calcium knee score, *KLG* Kellgren–Lawrence grade, *KOOS* knee injury and osteoarthritis outcome score, *DCE-MRI* dynamic contrast-enhanced magnetic resonance imaging, *IRE* initial rate of enhancement, *ME* maximum enhancement, *Nvoxel* most perfused voxels, *MOAKS* MRI osteoarthritis knee score, *BLOKS* Boston–Leeds OA knee score, *CE-synovitis* 11-point whole-knee synovitis score

^†^Independent sample *t*-test

^‡^ANCOVA

**Fig. 3:**
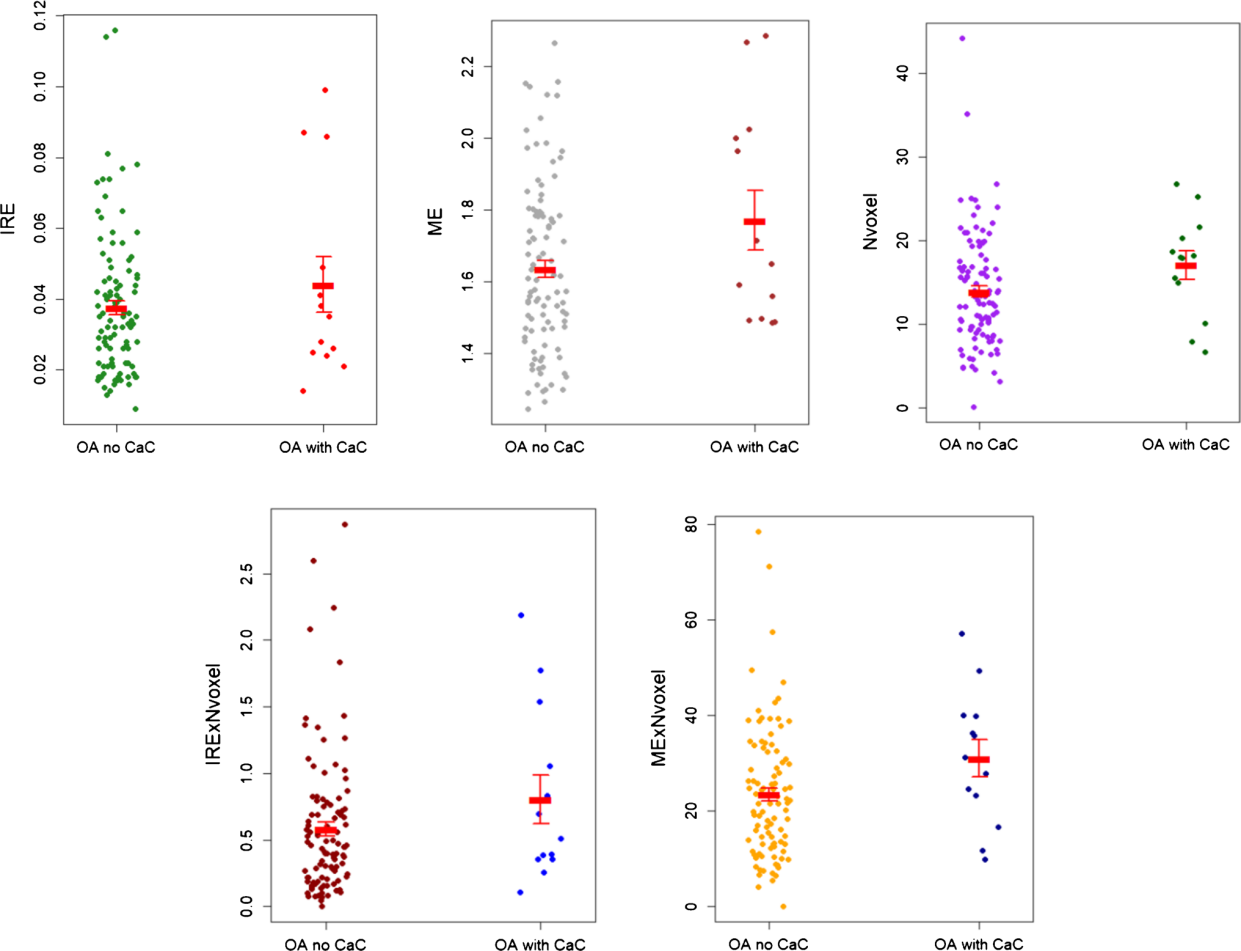
Strip plot of the DCE-MRI variables according to group. The red line represents the group mean with error bars (standard error). CaC Calcium crystal deposits, IRE initial rate of enhancement, ME maximum enhancement, Nvoxel most perfused voxels. OA no CaC, *n* = 102; OA with CaC, *n* = 13

Participants with MRI available had on average a lower BMI compared with the overall study population (34.6 (± 3.8) and 36.3 (± 5.4), respectively). Mean DCE and static MRI variables are presented in Table 1, along with the results from the ANCOVA analyses. None of the DCE-MRI variables were associated with the presence of CaCs, Fig. 3.

Mean (SD) KOOS-pain was 65.9 (± 17.2) for the OA with CaC group and 63.7 (± 16.4) for the OA without CaC group. We did not find a significant difference in mean KOOS-pain between the two groups; the mean difference was − 2.2 points (95% CI, − 10.86, 6.45), Table 1.

## Discussion

In this cross-sectional study, we explored if the presence of CaC deposits was associated with increased knee joint pain and MRI-derived parameters associated with joint inflammation in patients with overweight and knee OA. We did not find a significant difference in knee pain or MRI-detected inflammation between those participants with CaC deposits and those without CaC deposits.

Pain is the mainstay of patient-centered outcomes in OA research. However, pain perception in OA is complex. Structural disease severity assessed on imaging does not always correlate well with the clinical presentation of the disease, such as patient-reported pain and functional impairment [26]. In a study using the Osteoarthritis Initiative (OAI) dataset, knees with chondrocalcinosis had higher baseline and 4-year follow-up WOMAC pain scores compared to patients with OA without chondrocalcinosis on radiographs [10]. Conversely, a study using data from the Multicenter Osteoarthritis Study (MOST) did not find an association between intraarticular mineralization assessed on CT and WOMAC pain score but found a higher odds ratio of developing frequent knee pain in patients with knee OA and intraarticular mineralization [6]. In our study, the difference in KOOS-pain was small and did not reach statistical significance. Even though our study population was comparable to the two aforementioned studies on age and sex distribution, they only included participants with KLG ≤ 2 at baseline compared to a higher KLG in our study, where the majority of participants had KLG ≥ 2. This might explain the discrepancy between our findings. A study comparing patients with knee OA with patients with calcium pyrophosphate deposition disease (CPPD) found that the CPPD group had similar WOMAC pain scores to patients with mild OA, but patients with severe OA had worse WOMAC pain scores [27]. In our study, the small number of participants with CaC deposits did not allow sub-analyses stratified by disease severity, but crystal-induced pain may contribute less to the overall pain perception in patients with more severe OA.

Increased joint inflammation has been hypothesized as an underlying mechanism for increased pain and faster progression in patients with OA with CaC deposits [5]. However, a study assessing the association between chondrocalcinosis on radiographs and joint inflammation in the OAI dataset found no difference in MOAKS synovitis both at baseline and 4-year follow-up [10]. Another study found an increased odds ratio of having knee joint effusion in patients with CaCs assessed on ultrasound [9]. In the latter study, the authors found a high prevalence of sonographic CaCs: 73 of 106 knees; notably, only three patients had chondrocalcinosis on radiographs [9]. Furthermore, a study of patients with knee OA and knee effusion, assessing CPP crystals in SF, found an association between CPP crystals and inflammation in this subpopulation of patients with knee joint effusion [2]. This illustrates the challenge of the heterogeneity of the different imaging techniques used to detect CaC deposits. Patient populations vary among the available research, making comparison difficult. The EULAR recommendations on imaging in crystal-induced arthropathies published in 2024 highlight the variation of the available data on imaging techniques in this field [28]. We did not find a difference in joint inflammation between the two groups assessed by DCE-MRI. However, with only 13 participants with CaC, this could very well be due to a lack of power in our study. All mean DCE-MRI variables are higher in the OA with CaC group compared with the OA no CaC group, as illustrated in Fig. 3, although not significantly. As this was an exploratory analysis of an RCT dataset, it was not specifically designed for the present purpose, and our data house uncertainty of the estimates and large variance.

Other limitations of our study should be addressed. Firstly, CT was not available prior to weight loss intervention. However, we have not found reports to support a weight-loss-dependent decrease in CaCs. We are aware of one study on sequential SF analyses; they found that if CPP crystals were present in baseline SF, they were also present in all subsequent SF analyses [29]. Secondly, Patients who had MRI had a lower BMI compared to the overall study population due to the size constraints of the MRI coil. This could potentially impose a biased selection of patients with lower joint inflammation in the MRI analysis. However, in a study of the LOSEIT imaging data, significant weight loss did not induce significant changes in the MRI variables studied [17]. Finally, addressing the methodology used to detect CaC deposits is important. A limitation of CT, as with all non-invasive in vivo imaging modalities, is that it does not differentiate different calcium crystal types, so we can not speak to the role of CPP versus BCP. However, CT does have a higher sensitivity than radiographs [4] and BUCKS has shown high intra- and inter-reader reliability [14]. Another strength of our study is the use of DCE-MRI to assess knee joint inflammation. A recent meta-analysis found that DCE-MRI had a better correlation with microscopic synovitis compared to static CE-MRI, although on a limited sample size [30].

In conclusion, in this exploratory cross-sectional study, we did not find knee joint CaC deposits to be associated with knee pain or knee joint inflammation in individuals with overweight and knee OA.

## Supplementary Information

Below is the link to the electronic supplementary material.

Fig. S1 In the modified version of the Boston University Calcium Knee Score (BUCKS) used in the study, the hyaline cartilage is divided into 14 sub-regions as described in the Whole-Organ Magnetic Resonance Imaging Score (WORMS). The lateral and medial menisci are divided into three sub-regions: anterior horn, body, and posterior horn. For the 14 cartilage and 6 meniscal sub regions, an ordinal score of 0–3 is used for the degree of mineralisation of cartilage surface area or subregional meniscal volume. Grade 0 = no mineralisation, grade 1 = <10% mineralisation, grade 2 = 10–75% mineralisation, and grade 3 = >75%. Mineralisation of the joint capsule is graded 0 for absent or 1 for present. A–F: Sagittal reformats, mineralisation denoted with red arrows. A: Grade 1, mineralisation in the joint capsule. B: Grade 1, mineralisation in the posterior horn of the medial meniscus. C: Grade 2, mineralisation in the posterior horn of the lateral meniscus. D: Grade 3, mineralisation in the posterior horn of the medial meniscus. E: Grade 1, mineralisation in the posterior sub-region of the lateral femoral cartilage. F: Grade 2, mineralisation in the posterior sub region of the lateral tibial plateau cartilage. (PDF 270 KB)

## Data Availability

The data that support the findings of this study are available from the corresponding author (CTN) upon reasonable request.

## References

[1] Fuerst M, Bertrand J, Lammers L, Dreier R, Echtermeyer F, Nitschke Y, et al. Calcification of articular cartilage in human osteoarthritis. Arthritis Rheum [Internet]. 2009;60(9):2694–703. Available from: https://onlinelibrary.wiley.com/doi/10.1002/art.24774. 10.1002/art.24774 19714647

[2] Frallonardo P, Ramonda R, Peruzzo L, Scanu A, Galozzi P, Tauro L, et al. Basic calcium phosphate and pyrophosphate crystals in early and late osteoarthritis: relationship with clinical indices and inflammation. Clin Rheumatol [Internet]. 2018;37(10):2847–53. Available from: http://link.springer.com/10.1007/s10067-018-4166-3. 10.1007/s10067-018-4166-3 29882204

[3] Derfus BA, Kurian JB, Butler JJ, Daft LJ, Carrera GF, Ryan LM, et al. The high prevalence of pathologic calcium crystals in pre-operative knees. J Rheumatol [Internet]. 2002;29(3):570–4. Available from: http://www.ncbi.nlm.nih.gov/pubmed/11908575. 11908575

[4] Jarraya M, Guermazi A, Liew JW, Tolstykh I, Lynch JA, Aliabadi P, et al. Prevalence of intra-articular mineralization on knee computed tomography: the multicenter osteoarthritis study. Osteoarthr Cartil [Internet]. 2023;31(8):1111–20. Available from: https://linkinghub.elsevier.com/retrieve/pii/S1063458423007586. 10.1016/j.joca.2023.04.004 PMC10524737 37088266

[5] Foreman SC, Gersing AS, von Schacky CE, Joseph GB, Neumann J, Lane NE, et al. Chondrocalcinosis is associated with increased knee joint degeneration over 4 years: data from the osteoarthritis initiative. Osteoarthr Cartil [Internet]. 2020;28(2):201–7. Available from: https://linkinghub.elsevier.com/retrieve/pii/S1063458419312312. 10.1016/j.joca.2019.10.003 PMC7002267 31629813

[6] Liew JW, Jarraya M, Guermazi A, Lynch J, Wang N, Rabasa G, et al. Relation of intra‐articular mineralization to knee pain in knee osteoarthritis: a longitudinal analysis in the MOST study. Arthritis Rheumatol [Internet]. 2023;75(12):2161–8. Available from: https://acrjournals.onlinelibrary.wiley.com/doi/10.1002/art.42649. 10.1002/art.42649 PMC10770289 37410792

[7] Liu YZ, Jackson AP, Cosgrove SD. Contribution of calcium-containing crystals to cartilage degradation and synovial inflammation in osteoarthritis. Osteoarthr Cartil [Internet]. 2009;17(10):1333–40. Available from: https://linkinghub.elsevier.com/retrieve/pii/S1063458409001356. 10.1016/j.joca.2009.04.022 19447216

[8] Renaudin F, Orliaguet L, Castelli F, Fenaille F, Prignon A, Alzaid F, et al. Gout and pseudo-gout-related crystals promote GLUT1-mediated glycolysis that governs NLRP3 and interleukin-1β activation on macrophages. Ann Rheum Dis [Internet]. 2020;79(11):1506–14. Available from: https://linkinghub.elsevier.com/retrieve/pii/S0003496724011725. 10.1136/annrheumdis-2020-217342 32699039

[9] Mohammed RHA, Kotb H, Amir M, Di Matteo A. Subclinical crystal arthropathy: a silent contributor to inflammation and functional disability in knees with osteoarthritis—an ultrasound study. J Med Ultrason [Internet]. 2019;46(1):137–46. Available from: http://link.springer.com/10.1007/s10396-018-0912-z. 10.1007/s10396-018-0912-z 30327988

[10] Han BK, Kim W, Niu J, Basnyat S, Barshay V, Gaughan JP, et al. Association of chondrocalcinosis in knee joints with pain and synovitis: data from the osteoarthritis initiative. Arthritis Care Res (Hoboken) [Internet]. 2017;69(11):1651–8. Available from: https://acrjournals.onlinelibrary.wiley.com/doi/10.1002/acr.23208. 10.1002/acr.23208 PMC5529277 28129488

[11] Loeuille D, Sauliere N, Champigneulle J, Rat AC, Blum A, Chary-Valckenaere I. Comparing non-enhanced and enhanced sequences in the assessment of effusion and synovitis in knee OA: associations with clinical, macroscopic and microscopic features. Osteoarthr Cartil [Internet]. 2011;19(12):1433–9. Available from: https://linkinghub.elsevier.com/retrieve/pii/S1063458411002469. 10.1016/j.joca.2011.08.010 21930225

[12] Deveza LA, Melo L, Yamato TP, Mills K, Ravi V, Hunter DJ. Knee osteoarthritis phenotypes and their relevance for outcomes: a systematic review. Osteoarthr Cartil [Internet]. 2017;25(12):1926–41. Available from: https://linkinghub.elsevier.com/retrieve/pii/S1063458417311561. 10.1016/j.joca.2017.08.009 28847624

[13] Gudbergsen H, Overgaard A, Henriksen M, Wæhrens EE, Bliddal H, Christensen R, et al. Liraglutide after diet-induced weight loss for pain and weight control in knee osteoarthritis: a randomized controlled trial. Am J Clin Nutr [Internet]. 2021;113(2):314–23. Available from: https://linkinghub.elsevier.com/retrieve/pii/S0002916522005901. 10.1093/ajcn/nqaa328 33471039

[14] Guermazi A, Jarraya M, Lynch JA, Felson DT, Clancy M, Nevitt M, et al. Reliability of a new scoring system for intraarticular mineralization of the knee: Boston University calcium knee score (BUCKS). Osteoarthr Cartil [Internet]. 2020;28(6):802–10. Available from: https://linkinghub.elsevier.com/retrieve/pii/S1063458420309171. 10.1016/j.joca.2020.03.003 PMC8188576 32173626

[15] Roos EM, Lohmander LS. The knee injury and osteoarthritis outcome score (KOOS): from joint injury to osteoarthritis. Health Qual Life Outcomes [Internet]. 2003;1(64):1–8. Available from: https://hqlo.biomedcentral.com/articles/10.1186/1477-7525-1-64. 10.1186/1477-7525-1-64 PMC280702 14613558

[16] Roos EM, Roos HP, Lohmander LS, Ekdahl C, Beynnon BD. Knee injury and osteoarthritis outcome score (KOOS)—development of a self-administered outcome measure. J Orthop Sport Phys Ther [Internet]. 1998;28(2):88–96. Available from: http://www.jospt.org/doi/10.2519/jospt.1998.28.2.88. 10.2519/jospt.1998.28.2.88 9699158

[17] Daugaard CL, Henriksen M, Riis RGC, Bandak E, Nybing JD, Hangaard S, et al. The impact of a significant weight loss on inflammation assessed on DCE-MRI and static MRI in knee osteoarthritis: a prospective cohort study. Osteoarthr Cartil [Internet]. 2020;28(6):766–73. 10.1016/j.joca.2020.02.837. 10.1016/j.joca.2020.02.837 32165240

[18] Hunter DJ, Guermazi A, Lo GH, Grainger AJ, Conaghan PG, Boudreau RM, et al. Evolution of semi-quantitative whole joint assessment of knee OA: MOAKS (MRI osteoarthritis knee score). Osteoarthr Cartil [Internet]. 2011;19(8):990–1002. Available from: https://linkinghub.elsevier.com/retrieve/pii/S1063458411001531. 10.1016/j.joca.2011.05.004 PMC4058435 21645627

[19] Hunter DJ, Lo GH, Gale D, Grainger AJ, Guermazi A, Conaghan PG. The reliability of a new scoring system for knee osteoarthritis MRI and the validity of bone marrow lesion assessment: BLOKS (Boston–Leeds osteoarthritis knee score). Ann Rheum Dis [Internet]. 2008;67(2):206–11. Available from: https://linkinghub.elsevier.com/retrieve/pii/S0003496724219099. 10.1136/ard.2006.066183 17472995

[20] Guermazi A, Roemer FW, Hayashi D, Crema MD, Niu J, Zhang Y, et al. Assessment of synovitis with contrast-enhanced MRI using a whole-joint semiquantitative scoring system in people with, or at high risk of, knee osteoarthritis: the MOST study. Ann Rheum Dis [Internet]. 2011;70(5):805–11. Available from: https://linkinghub.elsevier.com/retrieve/pii/S0003496724145803. 10.1136/ard.2010.139618 PMC4180232 21187293

[21] Axelsen M, Stoltenberg M, Poggenborg R, Kubassova O, Boesen M, Bliddal H, et al. Dynamic gadolinium-enhanced magnetic resonance imaging allows accurate assessment of the synovial inflammatory activity in rheumatoid arthritis knee joints: a comparison with synovial histology. Scand J Rheumatol [Internet]. 2012;41(2):89–94. Available from: http://www.tandfonline.com/doi/full/10.3109/03009742.2011.608375 10.3109/03009742.2011.608375 22283139

[22] Vordenbäumen S, Schleich C, Lögters T, Sewerin P, Bleck E, Pauly T, et al. Dynamic contrast-enhanced magnetic resonance imaging of metacarpophalangeal joints reflects histological signs of synovitis in rheumatoid arthritis. Arthritis Res Ther [Internet]. 2014;16(5):452. Available from: https://arthritis-research.biomedcentral.com/articles/10.1186/s13075-014-0452-x 10.1186/s13075-014-0452-x PMC4201730 25270553

[23] Ballegaard C, Riis RGC, Bliddal H, Christensen R, Henriksen M, Bartels EM, et al. Knee pain and inflammation in the infrapatellar fat pad estimated by conventional and dynamic contrast-enhanced magnetic resonance imaging in obese patients with osteoarthritis: a cross-sectional study. Osteoarthr Cartil [Internet]. 2014;22(7):933–40. Available from: https://linkinghub.elsevier.com/retrieve/pii/S1063458414010565 10.1016/j.joca.2014.04.018 24821663

[24] Riis RGC, Gudbergsen H, Henriksen M, Ballegaard C, Bandak E, Röttger D, et al. Synovitis assessed on static and dynamic contrast-enhanced magnetic resonance imaging and its association with pain in knee osteoarthritis: a cross-sectional study. Eur J Radiol [Internet]. 2016;85(6):1099–108. 10.1016/j.ejrad.2016.03.017. 27161058 10.1016/j.ejrad.2016.03.017

[25] Cohen J. Statistical Power Analysis for the Behavioral Sciences [Internet]. 2nd ed. Routledge. 1988. Available from: https://www.taylorfrancis.com/books/9781134742707.

[26] Hunter DJ, Guermazi A, Roemer F, Zhang Y, Neogi T. Structural correlates of pain in joints with osteoarthritis. Osteoarthr Cartil [Internet]. 2013;21(9):1170–8. Available from: https://linkinghub.elsevier.com/retrieve/pii/S1063458413008224. 10.1016/j.joca.2013.05.017 23973127

[27] Whelan MG, Hayashi K, Altwies H, Tedeschi SK. Patient-reported outcomes in calcium pyrophosphate deposition disease compared to gout and osteoarthritis. J Rheumatol [Internet]. 2023;50(8):1058–62. Available from: http://www.jrheum.org/lookup/doi/10.3899/jrheum.2023-0031 10.3899/jrheum.2023-0031 PMC10496647 37061233

[28] Mandl P, D’Agostino MA, Navarro-Compán V, Geßl I, Sakellariou G, Abhishek A, et al. 2023 EULAR recommendations on imaging in diagnosis and management of crystal-induced arthropathies in clinical practice. Ann Rheum Dis [Internet]. 2024;83(6):752–9. Available from: https://linkinghub.elsevier.com/retrieve/pii/S0003496724001316. 10.1136/ard-2023-224771 PMC11103298 38320811

[29] Robier C, Neubauer M, Fritz K, Lippitz P, Stettin M, Rainer F. The detection of calcium pyrophosphate crystals in sequential synovial fluid examinations of patients with osteoarthritis: once positive, always positive. Clin Rheumatol [Internet]. 2013;32(5):671–2. Available from: http://link.springer.com/10.1007/s10067-012-2147-5. 10.1007/s10067-012-2147-5 23271610

[30] Shakoor D, Demehri S, Roemer FW, Loeuille D, Felson DT, Guermazi A. Are contrast-enhanced and non-contrast MRI findings reflecting synovial inflammation in knee osteoarthritis: a meta-analysis of observational studies. Osteoarthr Cartil [Internet]. 2020;28(2):126–36. Available from: https://linkinghub.elsevier.com/retrieve/pii/S1063458419312439. 10.1016/j.joca.2019.10.008 31678664

