# Supplementary figures and images for: The association between articular calcium crystal deposition and knee osteoarthritis, joint pain and inflammation: a cross-sectional study
Source: Skeletal Radiol. 2025 Mar 14;54(9):1939–47. doi: 10.1007/s00256-025-04904-7 (PMC12241293; doi:10.1007/s00256-025-04904-7)

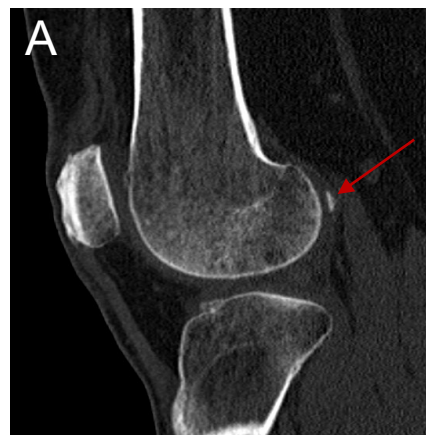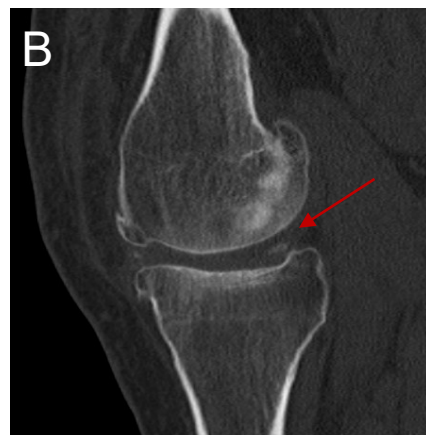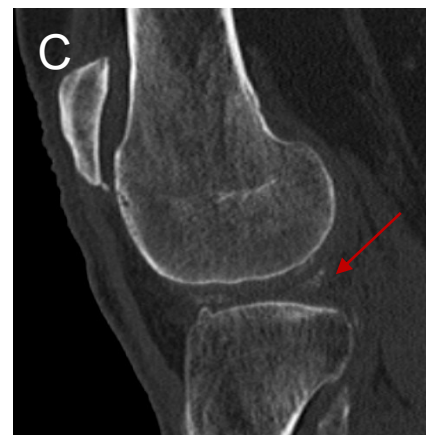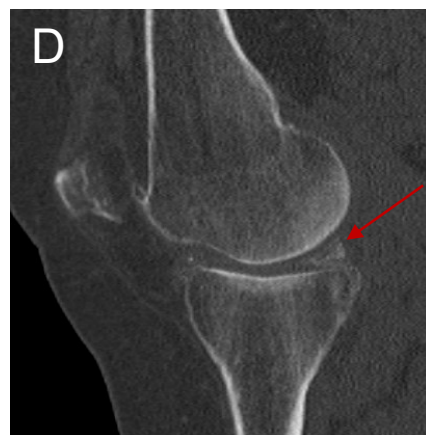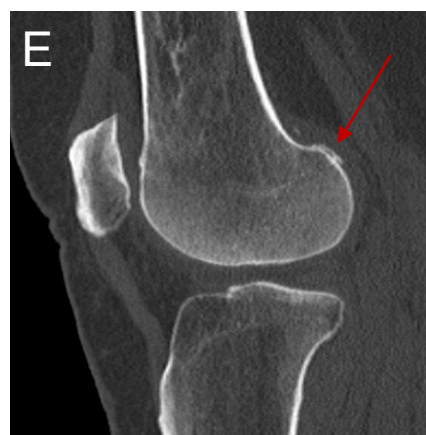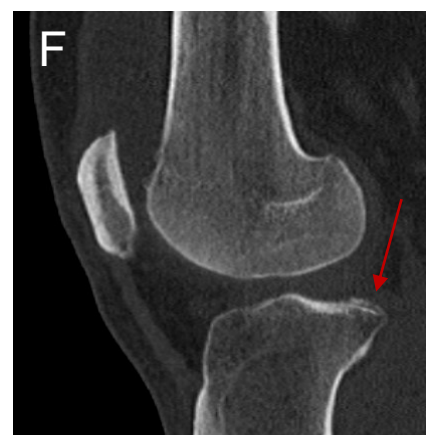

Supplement: Supplementary file 1 — In the modified version of the Boston University Calcium Knee Score (BUCKS) used in the study, the hyaline cartilage is divided into 14 sub-regions as described in the Whole-Organ Magnetic Resonance Imaging Score (WORMS). The lateral and medial menisci are divided into three sub-regions: anterior horn, body, and posterior horn. For the 14 cartilage and 6 meniscal sub regions, an ordinal score of 0–3 is used for the degree of mineralisation of cartilage surface area or subregional meniscal volume. Grade 0 = no mineralisation, grade 1 = <10% mineralisation, grade 2 = 10–75% mineralisation, and grade 3 = >75%. Mineralisation of the joint capsule is graded 0 for absent or 1 for present. A–F: Sagittal reformats, mineralisation denoted with red arrows. A: Grade 1, mineralisation in the joint capsule. B: Grade 1, mineralisation in the posterior horn of the medial meniscus. C: Grade 2, mineralisation in the posterior horn of the lateral meniscus. D: Grade 3, mineralisation in the posterior horn of the medial meniscus. E: Grade 1, mineralisation in the posterior sub-region of the lateral femoral cartilage. F: Grade 2, mineralisation in the posterior sub region of the lateral tibial plateau cartilage. (PDF 270 KB) [file 256_2025_4904_MOESM1_ESM.pdf]
